# Effect of an Extended Reality Simulation Intervention on Midwifery Students’ Anxiety: Systematic Review

**DOI:** 10.2196/68984

**Published:** 2025-06-18

**Authors:** Clara Pérez de los Cobos Cintas, Nicolas Vuillerme, Guillaume Thomann, Lionel Di Marco

**Affiliations:** 1 Laboratoire des Sciences pour la Conception, l'Optimisation et la Production, 46 Av. Félix Viallet, Grenoble, 38000, France, 33 04 76 57 50 71; 2 Autonomie, Gérontologie, E-santé, Imagerie et Société, 2Univ.Grenoble Alpes, AGEIS, Grenoble, France; 3 Department of Midwifery, Faculty of Medicine, Université Grenoble Alpes, France

**Keywords:** anxiety, extended reality, gesture simulation, nursing education, midwifery education

## Abstract

**Background:**

Midwifery students often experience anxiety due to several factors, such as the clinical experiences faced. Simulation-based learning in nursing and midwifery studies using extended reality (XR) tools offers the opportunity to manage better educational processes while reducing this anxiety.

**Objective:**

This study aims to evaluate the current knowledge and understanding of how the use of XR gesture-simulation-based tools allows a better understanding of the anxiety levels of midwives and nurses in educational settings.

**Methods:**

We conducted a systematic review, a scientific literature search following the PRISMA (Preferred Reporting Items for Systematic Reviews and Meta-Analyses) guidelines. Using PubMed, IEEE, Scopus, and Web of Science, up to March 2024, 1005 articles were found to identify studies that reported the effectiveness of these technologies for gesture simulation in education and training on nursing and midwifery student anxiety. The inclusion-exclusion criteria were based on the PICO (population, intervention, control, and outcomes) framework. The population included nurses, midwives, and nursing and midwifery students of any kind using any virtual or augmented or mixed reality simulation training tool to perform a procedure aimed at reducing anxiety. In addition, the Cochrane risk of bias tool was used to evaluate the quality of the systematic review and the bias in the included studies. A narrative synthesis was conducted due to the heterogeneity of study designs and outcome measures. Key findings were summarized in a structured table and grouped according to the learning objective, simulating and performing procedures in an educational setting.

**Results:**

Overall, 7 articles, involving a total of 428 participants, were included in this review. The findings indicate that XR can effectively reduce anxiety in midwifery and nursing education. However, the limited number of studies highlights a research gap in the field, particularly in the area of mixed reality, which warrants further exploration.

**Conclusions:**

This systematic review highlights the potential of XR-based gesture-simulation tools in reducing anxiety among midwifery and nursing students. The included studies suggest that XR-enhanced training provides a more immersive and controlled learning environment, helping students manage stress and improve procedural confidence. However, the limited number of studies, methodological variations, and the underrepresentation of mixed reality applications indicate the need for further research. Future studies should focus on standardized anxiety measurement tools, larger sample sizes, and long-term impact assessments to strengthen the evidence base. Expanding research in this field could enhance the integration of XR technologies into midwifery and nursing education, ultimately improving both learning experiences and clinical preparedness.

## Introduction

Anxiety is an emotion or state characterized by worry, tension, and physical manifestations such as increased blood pressure. Anxiety is often experienced in midwifery students due to heavy course load, high expectations, and the clinical experiences faced [1].

According to a mental health survey conducted by the National French Association of Midwifery Students (ANESF), with responses from 2000 students, 47% of the respondents showed a probable generalized anxiety disorder (as indicated by the GAD-7 test, General Anxiety Disorder-7, with a score of ≥10) [2,3].

Simulation-based learning is an important education modality because it allows students to learn from mistakes in a risk-free setting, acquire necessary competencies, practice decision-making, and significantly reduce potentially fatal medical errors [4]. More precisely, through gesture simulation, students can interact with virtual reality (VR) environments using hand movements and gestures, thereby reinforcing muscle memory and enhancing kinesthetic learning. This hands-on approach enables students to better grasp complex concepts, refine their motor skills, and simulate real-world scenarios with greater accuracy. In addition, gesture simulation fosters active engagement and participation, allowing students to feel more connected to the learning process and empowering them to take ownership of their education [5].

Extended Reality (XR) is a general term encompassing a range of immersive technologies, including Augmented Reality (AR), Virtual Reality (VR), Mixed Reality (MR):

1. AR overlays digital information, such as images, text, or 3D models onto the real-world environment viewed through a device’s camera. The digital content is superimposed on the real world, enhancing the user’s perception of reality [6].
2. VR completely immerses the user in a synthetic digital environment, replacing the real world with a simulated one. Users experience this artificial 3D environment through a headset that blocks out their physical surroundings [7].
3. MR seamlessly blends and anchors digital objects into the real world, allowing users to interact with physical and VR elements in real time. The VR objects in MR are integrated into and responsive to the real environment, creating a unified, interactive experience [8].

These tools blend physical and VR environments and over the last years, they have been in many fields, such as health care education or professional contexts, since they have the potential advantage of scalability, enhanced motivation, and cost savings [9].

By introducing XR simulation tools in midwifery education, the students can better understand complex concepts, practice skills in a safe environment, and build confidence in their abilities, reducing anxiety-inducing situations [10]. However, there is relatively limited research on the specific impact of these tools on factors such as confidence, anxiety, or decision-making capacities. Most studies focus primarily on performance outcomes or knowledge acquisition. Furthermore, while simulation in general has been extensively studied, research specifically addressing the use of XR tools in these contexts remains scarce [11]. This review aims to address these gaps by systematically analyzing the effectiveness of XR gesture-simulation tools in analyzing anxiety levels during educational training for midwives and nurses.

## Methods

### Research Question

The first step was the formulation of the research question, aiming to explore the impact of extended reality gesture-simulation-based tools on the anxiety levels of midwives and nurses involved in educational training. The research question “How much do we know about the effectiveness of extended reality gesture-simulation-based tools on the anxiety levels of midwives and nurses involved in educational training?” was formulated.

To answer the question, we performed the systematic review using the PICO (population, intervention, control, and outcomes) framework, which is an evidence-based practice to frame and answer a scientific endeavor [12]. The components of the framework applied in our study are as follows:

1. Population: Midwives and nurses of any type
2. Intervention: Extended reality gesture simulation-based training or simulation education intervention aimed at reducing anxiety
3. Comparison: Standard training methods or absence of specific anxiety-reduction interventions. Pre and postintervention anxiety levels
4. Outcome: Assessing anxiety levels or anxiety-related outcomes

The second category of keywords, related to the intervention, included: ”extended reality” OR ”XR” OR ”augmented reality” OR ”AR” OR ”virtual reality” OR ”VR” OR ”mixed reality” OR ”3D.”The third and last category was related to the desired outcome, including: ”anxiety” OR ”anxious.” The combination of all these keywords resulted in the final query:

(“midwife” OR “obstetrics” OR “nurse” OR “nursing”) AND (“mixed reality” OR “MR” OR “extended reality” OR “XR” OR “augmented reality” OR “AR” OR “virtual reality” OR “Simulation” OR “Simulated” OR “3D”) AND (“anxiety” OR “anxious”)

### Screening Criteria

Different search tools, including PubMed, IEEE, Scopus, and Web of Science, were used to perform the systematic research with the previously defined keywords. The database search and extraction were performed on March 25, 2024. Besides, the articles retrieved from another similar systematic review were analyzed to see whether they could be included [13].

In total, 1005 articles were found; the decision to include or exclude a study was based on the inclusion and exclusion criteria and quality assessment. A single author screened the articles, and the selection process and rationale were subsequently discussed with the other authors to finalize inclusion. Any disagreement between the reviewers regarding inclusion was resolved with discussion and a majority vote. After applying these criteria, 7 articles were selected for the review.

### Search Strategies

The search strategy focused on databases most relevant to health care and XR research, including PubMed, IEEE, Scopus, and Web of Science. These information sources were selected to ensure coverage of both medical and technological aspects of XR interventions in anxiety analysis during midwifery and nursing gesture simulation activities. Gray literature and conference proceedings were excluded due to resource constraints and difficulty in quality assessment. By not including gray literature and unpublished studies, there is a risk of publication bias, as studies with nonsignificant findings may be underrepresented.

A PubMed MeSH search was conducted to ensure the inclusion of relevant studies using the previously identified keywords to enhance the completeness of the findings. MeSH (Medical Subject Headings) is the National Library of Medicine’s controlled vocabulary thesaurus, used for indexing articles for the MEDLINE/PubMED database [14]:

((“students, nursing”[MeSH Terms] OR “Obstetric Nursing”[MeSH Terms] OR “nursing students”[Title/Abstract] OR “midwifery”[MeSH Terms] OR “midwifery students”[Title/Abstract]) AND (“virtual reality”[MeSH Terms] OR “augmented reality”[Title/Abstract] OR “extended reality”[Title/Abstract]) AND (“anxiety”[MeSH Terms] OR “anxious”[Title/Abstract]))

A total of 3 articles were found, the first one was focused on the discovery and visit to places, the second was a systematic review, and the third used VR to reduce anxiety by singing calming environments. Therefore, none of the articles were included in this systematic review.

### Relevance and Topic Proximity: Inclusion and Exclusion Criteria

The PRISMA (Preferred Reporting Items for Systematic Reviews and Meta-Analyses) methodology was used to search and screen articles [15]. In addition, the PRISMA checklist was completed to provide transparency and rigor in the systematic review process, in Checklist 1.

The initial choice was to narrow down the results by only selecting articles in English published in a peer-reviewed journal, randomized controlled trials (RCTs), nonrandomized, noncontrolled trials.

The second selection phase encompassed screening titles, keywords, and abstracts, followed by the removal of duplicate articles. Throughout the process, the inclusion and exclusion criteria explained in Table 1 were applied.

**Table 1.** Inclusion and exclusion criteria for article screening.

Element of the research question	Inclusion criteria	Exclusion criteria
Population	Nurses, midwives, and nursing and midwifery students of any kind	Any type of health care professional or other students, any other population such as patients
Intervention	Any virtual or augmented or mixed reality simulation training tool to perform a procedure (VR^a^ glasses such as HoloLens, Oculus Rift, or any other type of device as the CAVE)^b^ used to reduce *anxiety*	Any other tool or simulator not used in this setting
Comparator	XR^c^ simulation vs other standard training methods, or pre and postintervention anxiety levels	N/A^d^
Outcome	Anxiety assessment	N/A
Study design, type of publication	Articles in English, French, and Spanish published in a peer-reviewed journal, randomized controlled trials (RCTs), non-randomized, noncontrolled trials	Journal articles in other languages, conference papers, book chapters, reviews, meta-analysis pilot studies, proof of concept

a VR: virtual reality.

b CAVE: Cave Automatic Virtual Environment.

c XR: extended reality.

d N/A: Not available

### Quality Assessment

The Cochrane Collaboration’s Risk of Bias 2.0 tool [16,17] was used to evaluate the quality of the systematic review and assess the risk of bias in the included studies. The tool assesses five specific domains of bias: bias in the randomization process, bias in deviation from intended interventions, bias due to missing outcome data, bias in outcome measurement, and bias in the selection of the reported result.

Items were classified as “Low risk,” “High risk,” or “Some concerns.” The overall risk was “Low risk” if all five domains were rated as low risk, “High risk” if any domain was rated as high risk, and “Some concerns” for all other cases.

### Result Analysis

To extract and analyze the resulting articles, data was extracted and collected systematically in a data collection form. A narrative synthesis was conducted due to the heterogeneity of study designs and outcome measures across included studies. Key findings were summarized in a structured table and grouped according to the learning objective (simulating and performing procedures using XR tools). A thematic analysis was used to identify common patterns in anxiety analysis across studies.

## Results

### Summary of the Chosen Articles

**Figure 1.**
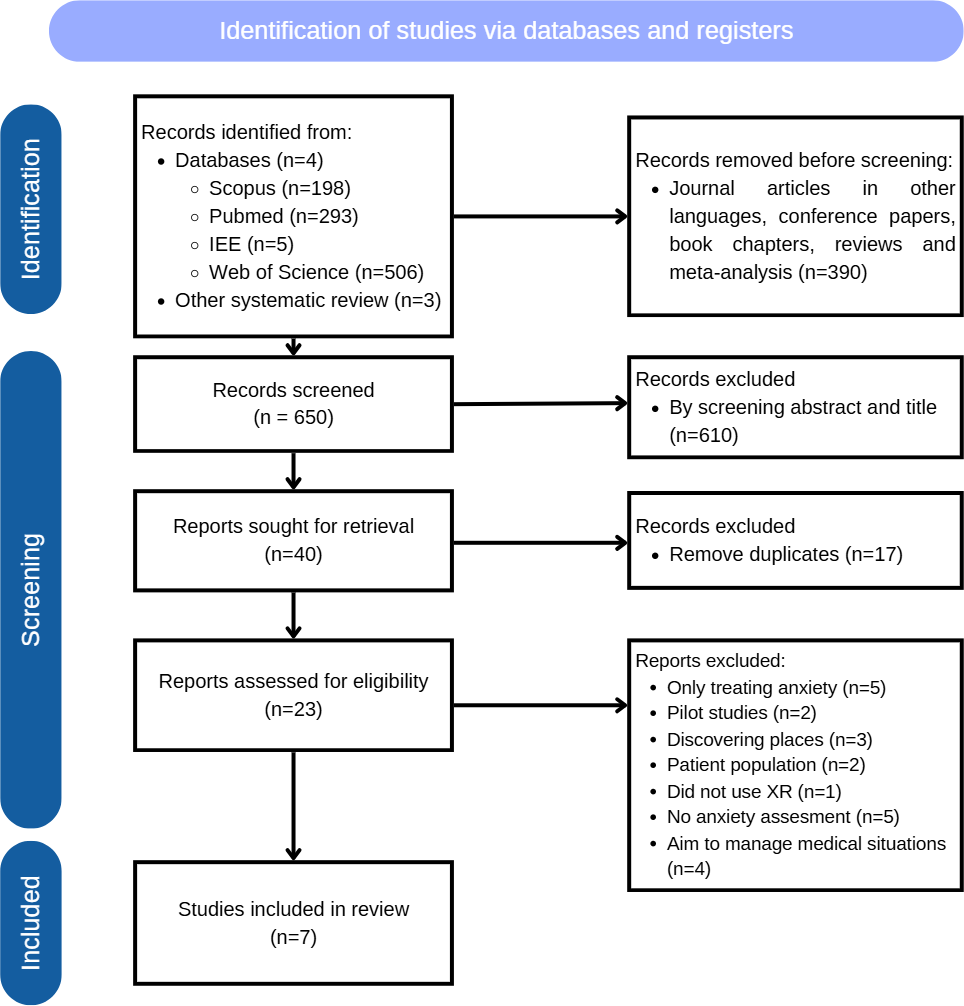
PRISMA flow diagram. XR: extended reality.

The systematic review of the literature is outlined in Figure 1. Table 2 and Table 3 summarize the results of the systematic review. Below is a brief summary of each of the articles included in the review.

**Table 2.** Systematic review results.

Ttitle	Year	Author	Journal	Location	Risk of bias	Learning object	Study design / Intervention assignment	Population
The effects of neonatal resuscitation gamification program using immersive virtual reality: A quasi-experimental study	2022	Yang and Oh [18].	Nurse Education Today	Daejeon, South Korea	Some concern	Simulating and performing procedures	Pre and postcomparison, non randomized study	Nursing students (n=88)
Immersive virtual reality (VR) training increases the self-efficacy of in-hospital healthcare providers and patient families regarding tracheostomy-related knowledge and care skills A prospective pre-post study	2022	Chiang et al [19].	Medicine Open	Taipei, Taiwan	Some concern	Simulating and performing procedures	Prospective, randomized controlled, pre and postcomparison study	Health care providers (n=60)
Pediatric Chest Compression Improvement Via Augmented Reality Cardiopulmonary Resuscitation Feedback in Community General Emergency Departments: A Mixed-Methods Simulation-Based Pilot Study	2023	Kleinman et al [20].	The Journal of Emergency Medicine	Baltimore, United States	Some concern	Simulating and performing procedures	Unblinded, randomized, crossover simulation-based study	Nurses (n=36)
Effects of virtual reality training on decreasing the rates of needlestick or sharp injury in new-coming medical and nursing interns in Taiwan	2020	Wu et al [21].	Journal of Educational Evaluation for Health Professions	Taipei, Taiwan	Some concern	Simulating and performing procedures	Prospective cohort pre and postcomparison study	Medical and Nurses Interns (n=109)
A mixed-methods feasibility study to assess the acceptability and applicability of immersive virtual reality sepsis game as an adjunct to nursing education	2021	Adhikari et al [22].	Nurse Education Today	Edinburgh, Scotland	Some concern	Simulating and performing procedures	Two-stage sequential mixed-methods feasibility study	Nursing students (n=19)
Virtual versus face-to-face clinical simulation in relation to student knowledge, anxiety, and self-confidence in maternal-newborn nursing: A randomized controlled trial	2016	Cobbett and Snelgrove-Clarke [23].	Nurse Education Today	Halifax, Canada	Some concern	Simulating and performing procedures	Controled, randomized pretest-posttest study	Nursing students (n=56)
Evaluation of practical exercises using an intravenous simulator incorporating virtual reality and haptics device technologies	2012	Jung et al [24].	Nurse Education Today	Seoul, Korea	Some concern	Simulating and performing procedures	Randomized control trial	First-year nursing students (n=60)

**Table 3.** Study characteristics vs study intervention characteristics

Studies	Software and devices	Intervention activities/ duration	Variables	Intervention outcomes	Anxiety evaluation
Yang and Oh [18]	HMD:^a^ Oculus Rift S (VR^b^)	Neonatal resuscitation gamification-50 min	Knowledge, problem-solving, clinical reasoning ability, self-confidence, anxiety, and learning motivation	Anxiety decreased post intervention, no difference between groups	STAI^c^
Chiang et al [19]	HMD-VR Web-based VR Desktop Tablet Smartphone	Tracheostomy-related knowledge and care skills-2-h training; 15-min VR	Familiarity Confidence Anxiety Knowledge Skills	Improved comprehension and reduced anxiety at follow-up	Personalized Likert scale questionnaires
Kleinman et al [20]	HMD (AR^d^)	Pediatric chest compressions-18 min course	Performance and anxiety (qualitative assessment)	Reduced anxiety	Qualitative invdividual interviews post intervention
Wu et al [21]	Game-based VR training	Training on needlestick or sharp injury time to finish the game	Performance and Anxiety (qualitative assesment)	Anxiety reduction, increase in confidence	Personalized Likert scale questionnaires
Adhikari et al [22]	3D computer-based simulation (VR)	Sepsis serious game - 20‐30 min	Anxiety (NASC-CDM)^e^, self-efficacy, acceptability, and applicability	Decreased anxiety	NASC-CDM before and after intervention
Cobbett and Snelgrove-Clarke [23]	F2F^f^ high-fidelity manikin simulation. VCS^g^ with a computer.	Newborn nursing - 2×45 min VR sessions	Simulation Completion Questionnaire, anxiety (NASC-CDM), knowledge	Higher anxiety for VR	NASC-CDM before and after intervention
Jung et al [24]	VR IV^h^ training simulator using haptic skills.	Venipuncture training-10 min session	State trait anxiety, VAS^i^, performance, and satisfaction	No difference between groups and anxiety decreased for all of them	Evaluated State-Trait Anxiety using a VAS before and after intervention

a HMD: head-mounted device

b VR: virtual reality

c STAI : State-Trait Anxiety Inventory

d AR: augmented reality

e NASC-CDM: Nursing Anxiety and Self-Confidence with Clinical Decision-Making Scale

f F2F: face-to-face

g VCS: Virtual Clinical Simulation

h IV: intravenous

i VAS: Visual Analogue Scale

### Short Summary of Each Article

#### The Effects of Neonatal Resuscitation Gamification Program Using Immersive Virtual Reality: A Quasi-Experimental Study

This nonrandomized controlled simulation study with a pretest-posttest design evaluated a neonatal resuscitation gamification program using immersive VR [18]. Prelicensure nursing students were divided into intervention and control groups. The study assessed outcomes such as neonatal resuscitation nursing knowledge, problem-solving skills, clinical reasoning ability, self-confidence in practical performance, anxiety levels, and learning motivation. The simulation group presented lower anxiety levels, compared to the VR and control groups. Anxiety was measured with the STAI (State-Trait Anxiety Inventory), a commonly used questionnaire to assess an individual’s tendency to suffer anxiety. Limitations include the inability to assess long-term effects through follow-up surveys and the lack of measurement for actual intervention competency reinforcement, relying solely on self-reported questionnaires.

#### Immersive VR Training Increases the Self-Efficacy of In-Hospital Health Care Providers and Patient Families Regarding Tracheostomy-Related Knowledge and Care Skills a Prospective Pre-Post Study

This prospective pre-post study compared health care providers’ tracheostomy care training using immersive VR with head-mounted displays and web-based modules versus traditional text materials [19]. According to a personalized Likert-scale questionnaire, most providers in the VR group found that interactive visual demonstrations improved comprehension and reduced anxiety. Limitations included a small sample size, a short follow-up period, and reliance on self-reported feedback rather than quantitative measures of skill acquisition and patient outcomes. Larger, long-term studies with objective assessments are needed to evaluate the efficacy of VR training in improving tracheostomy care competency.

#### Pediatric Chest Compression Improvement Via Augmented Reality Cardiopulmonary Resuscitation Feedback in Community General Emergency Departments: A Mixed-Methods Simulation-Based Pilot Study

An unblinded, randomized, crossover simulation-based study evaluated whether augmented reality cardiopulmonary resuscitation (AR-CPR) improves chest compression performance in nonpediatric-specialized community emergency departments [20]. Participants performed chest compression with and without AR-CPR guidance in random order. Qualitative interviews suggested AR-CPR could be usable without device orientation, effective at cognitive offloading, and capable of reducing anxiety while boosting confidence. However, limitations included not excluding individuals with corrective eye lenses, which might affect the AR experience and the lack of feedback during non-AR-CPR cycles, unlike real-world scenarios where feedback devices are common.

#### Effects of Virtual Reality Training on Decreasing the Rates of Needlestick or Sharp Injury in New-Coming Medical and Nursing Interns in Taiwan

The prospective cohort pre and post study evaluated a new VR game, designed to teach safe and unsafe behaviors regarding universal precautions on needlestick and sharp injury prevention among incoming medical and nursing interns in Taiwan [21]. The game focused on making correct safety choices. Many participants reported reduced anxiety about preventing these injuries in the personalized Likert-scale questionnaires. However, the study’s reliance on self-reported questionnaires could introduce reporting bias, and trainees might report behaviors aligning with their training, potentially skewing results.

#### A Mixed-Methods Feasibility Study to Assess the Acceptability and Applicability of Immersive Virtual Reality Sepsis Game as an Adjunct to Nursing Education

A 2-stage sequential mixed-methods feasibility study assessed the impact of an immersive VR sepsis game on preregistration nurses [22]. The study examined its effect on self-efficacy and perceptions of its acceptability and applicability in nursing simulation education. In the first stage, pre and postintervention self-efficacy scores were collected from 19 preregistration nurses using the Nursing Anxiety and Self-Confidence with Clinical Decision-Making Scale (NASC-CDM). The second stage used a descriptive qualitative approach to explore perceptions of the game. Results showed a significant 23.4% decrease in anxiety. Limitations included the small sample size, the novelty of the educational approach, and the measurement of self-efficacy at a single time point.

#### Virtual Versus Face-To-Face Clinical Simulation in Relation to Student Knowledge, Anxiety, and Self-Confidence in Maternal-Newborn Nursing: A Randomized Controlled Trial

This randomized pretest-posttest study compared the effectiveness of 2 maternal newborn clinical simulation scenarios: VR clinical simulation and face-to-face high-fidelity manikin simulation [23]. Although no statistically significant differences were found in student knowledge and self-confidence between the 2 modalities, anxiety scores, measured by the NASC-CDM, were higher for students in the VR simulation. Limitations included a small sample size, potential intervening variables, such as student motivation, interest, and technological competence, and a lack of orientation to the VR platform, which may have influenced their responses.

#### Evaluation of Practical Exercises Using an Intravenous Simulator Incorporating Virtual Reality and Haptics Device Technologies

This randomized control trial assessed the educational effectiveness of practical exercises using intravenous simulators with VR/haptics technologies [24]. First-year nursing students were assigned randomly to three groups: Group A (conventional intravenous arm), Group B (VR/haptics intravenous simulator), and Group C (both intravenous arm and simulator). Group C scored highest in venipuncture procedures, while Group B excelled in injections. Group C completed venipuncture faster than Group B and slightly quicker than Group A. State-trait anxiety was measured using a Visual Analogue Scale (VAS) before and after the intervention. All groups showed reduced anxiety postvenipuncture, with no significant differences. Limitations included insufficient practice time due to curriculum constraints and the study’s single-school, single-country setting, which limits generalizability and cultural diversity.

### Analysis of the Chosen Articles

#### Risk of Bias

After analyzing the risk of bias of the 7 articles included in the systematic review, summarized in Table 4, 1 [18] had a high risk in the randomization process because the participants were assigned systematically to the intervention and control groups. In total, 2 studies [21,22] lacked a control group and conducted pre and postintervention evaluations. The remaining 4 studies [19,20,23,24] demonstrated a low risk of bias.

**Table 4.** Risk of bias of the included studies.

Author	Year	Randomization process	Deviations from intended interventions	Missing outcome data	Measurement of outcome data	Selection of the reported result	Overall bias
Yang et al [18].	2022	High risk	Low risk	Low risk	Some concern	Low risk	Some concern
Chiang et al [19].	2022	Low risk	Some concern	Low risk	Low risk	Low risk	Some concern
Kleinman et al [20].	2023	Low risk	Low risk	Low risk	Some concern	Low risk	Some concern
Wu et al [21].	2020	Not applicable	Low risk	Low risk	Some concern	Low risk	Some concern
Adhikari et al [22].	2021	Not applicable	Low risk	Low risk	Some concern	Low risk	Some concern
Cobbett and Snelgrove-Clarke [23].	2016	Low risk	Some concern	Low risk	Low risk	Low risk	Some concern
Jung et al [24].	2012	Low risk	Some concern	Low risk	Low risk	Low risk	Some concern

The risk of deviations from the intended intervention raised some concerns in 3 studies due to the motivation of the intervention group related to their interest in the technology [19,23,24]. No risk was detected due to missing outcome data. However, some concerns arose in studies [18,20,21], regarding outcome measurement, as only the intervention group was evaluated in certain aspects. Finally, no risk was identified in any of the studies concerning the selection of reported results. In conclusion, all studies presented some concerns, but none was deemed to have a high risk of bias overall.

#### Learning Object

All of the papers had a common general learning objective: simulating and performing procedures. However, the clinical foci covered in the selected papers differed.

Overall, 2 of the studies focused on reducing anxiety around newborn care. The first was with an immersive VR neonatal resuscitation gamification program, enabling hands-on experience in a VR environment [18]. The second, a simulation-based pilot study used AR to teach pediatric chest compression [20].

In total, 2 studies used serious games to teach and reduce anxiety in students. Occupational needle stick or sharp injury prevention was sought through a game of right and wrong choices for safe or unsafe universal precaution behaviors [21]. The other used a 3D, computer-based simulation sepsis game [22].

In 3 studies, they opted for a procedure-specific training, Cobbett and Snelgrove-Clarke [23], evaluated VR clinical simulation in preeclampsia and Group B *Streptococcus* scenarios. Another study divided the first-year nursing students into 3 groups, learning how to practice injections. A VR intravenous training simulator using haptic skills was evaluated, along with its effect on reducing students’ anxiety toward this procedure [24]. Finally, in [19] used a combination of head-mounted display VR and smartphone application VR in an environment for tracheostomy-related materials. The VR teachings on how to dress the stoma or handle emergencies such as aspiration, aimed to reduce anxiety among health care providers.

#### Study Design

This analysis reveals significant methodological differences among the articles, particularly in their approaches to study design, including quasi-experimental, randomized controlled trials (RCTs), mixed methods, pre-post, and crossover designs.

RCTs ([20,23,24]) provide stronger evidence due to their randomization and ability to control the relationship between the XR interventions and outcomes. Quasi-experimental ([18]) and pre-post ([20,22,23]) designs are valuable for exploratory or feasibility research, but are less robust due to biases and limitations in establishing causality. Mixed-methods designs ([20,22]) add depth to the analysis by incorporating qualitative perspectives but lack the quantitative generalizability of RCTs. Finally, crossover designs ([20]) offer a unique advantage by enabling within-subject comparisons, which may result in more reliable conclusions about XR effectiveness.

The studies demonstrate that the choice of study design is dependent on the research goals, feasibility, and the need to balance internal validity, external validity, and depth of analysis. RCTs are the most robust and reliable for establishing causality, making them the gold standard for intervention studies. However, quasi-experimental designs and cohort studies provide practical alternatives for real-world contexts where randomization is not feasible. Meanwhile, mixed-methods designs offer depth and insights into user experiences, making them ideal for exploratory and feasibility studies, though their findings may be less generalizable. Finally, crossover designs effectively reduce variability and improve validity but require careful management to avoid carryover effects.

#### Participants

All the selected articles included nurses, midwives, or nursing and midwifery students of any kind. In total, 428 individuals participated in the studies, and 332 were nursing students, mixed with other medical students. Only 2 studies included 96 professional health care providers [19,20].

Exclusion criteria varied across the studies. In one case, they excluded those with previous clinical or VR experience, recruiting 88 prelicensure nursing students (VR group=31, simulation group=28, control group=29) [18]. The groups were homogeneous in sex, satisfaction with the nursing major, clinical practice training, and demand for XR education, but differed in anxiety levels.

Notably, 2 studies included third-year nursing students, with 56 and 19 participants, respectively [22,23]. The age ranges and gender proportions were similar: 20‐44 years, mostly female (84%), with no previous degree (81%) in the former study, and 25‐45 years, with 74% female in the latter. No statistical differences were found between the types of simulation groups.

One invited both 50 medical and 59 nursing interns [21]. Nursing interns were aged 17‐22 years, and medical interns were 20‐29 years. Female representation was 85% in nursing and 52% in medicine. The previous deep occupational experience was 34% in nursing and 61% in medicine.

In one study, 36 professional nurses (18 per group, AR and no AR) evenly distributed by age, sex, clinical role, and experience participated, with participants excluded based on their medical specialty [20]. Also involving 60 professional health care providers, including physicians, nurses, and respiratory therapists, in the study they randomly divided participants into regular and intervention groups, excluding those with incomplete training or questionnaires [19]. A similar exclusion criteria was applied to the same number of nursing students, ensuring homogeneity in age, gender, anxiety, and intravenous knowledge [24].

#### Devices Used to Create the Extended Reality Scenarios and Duration of the Intervention

In total, 6 articles used VR interventions, while one used AR [20]. Among included studies, 3 used a Head-Mounted device [18-20], 3 relied on computer-based simulations [22-24], and 1used a mobile device app.

The duration of the training sessions varied minimally across the studies, ranging from a 10-minute VR session [24], to a 50-minute gamification program [18]. Notably, only one conducted two sessions [23], while the others performed a single XR experiment.

#### Analysis and Anxiety Assessment Methods, Variables Evaluated

Throughout the studies, common elements were analyzed. The 7 articles evaluated anxiety using various methods, such as personalized Likert scale questionnaires [19,21]. Among them, 2 of the articles [22,23] useda specific tool to measure anxiety pretest and posttest, the NASC-CDM, which aims to provide insight into the emotional aspects of clinical decision-making, which can impact nursing performance and patient care outcomes [25].

A total of 2 studies assessed state-trait anxiety. One study distinguished between temporary anxiety influenced by environmental factors and the more stable, underlying trait anxiety [24]. Transient anxiety was measured using a Visual Analogue Scale, which consists of a 10-cm horizontal line with marked points corresponding to different levels of worry, such as “Not worried at all,” “Worried a little bit” and “Very worried,”. The chosen point on the VAS was converted to a numerical score for pre and posttest comparison. This analysis was performed and compared pre and post test. The other study [18] used the STAI, a 20-item questionnaire assessing state anxiety. Each item is rated on a 4-point Likert scale (1=not at all; 4=very much so), with a total score ranging from 20 to 80 (not 15 to 75 as stated in the original). Scores of 30 or lower indicate low or no anxiety, while scores of 31 or higher indicate high anxiety. The STAI’s Cronbach α was 0.93 at its development and 0.90 in this study.

Finally, in [20] they assessed anxiety by performing qualitative individual interviews while evaluating the rate and depth of chest compressions to provide AR feedback and therefore measure performance simultaneously.

Other aspects of the studies were evaluated, including knowledge pre and post test related to preeclampsia and group B strep, a Simulation Completion Questionnaire [23], knowledge related to neonatal resuscitation [18], or an multiple choice questionnaire on tracheostomy care and skills [19]. Another qualitative approach explores student nurses’ perceptions of the game [22], while others evaluated only the performance of the subjects, regardless of the knowledge acquisition [20,21].

#### Anxiety Outcomes After XR Intervention

After assessing and analyzing the results, most of the articles reported positive outcomes concerning anxiety reduction, as seen in the summary of anxiety outcomes in Table 5.

**Table 5.** Anxiety outcomes in the included studies.

*Autho*r	Intervention	Assessment tool	Population	Outcome
Yang et al [Bibr R17][18]	Intervention vs control; pre vs post test	STAI^a^	N=88; Intervention=31	Intervention: 59.14 (SD 9.62) to 56.72 (SD 7.50) Control: 59.50 (SD 8.35) to 57.65 (SD 6.86)
Chiang et al[Bibr R18] [19]	Intervention vs control; pre vs post test	Likert scale	N=60 ; Intervention=30	Intervention: reduced anxiety (mean 93%, SD 2%; *P*=.002 Control: reduced anxiety (mean 75% SD 6%; *P*=.002
Kleinman et al [Bibr R19][20]	Interview postintervention	Qualitative interviews	N=36	Qualitative answers about decrease of anxiety
Wu et al[Bibr R20] [21]	Pre- post-intervention	Likert scale questionnaire	N=109	63.3% reported a decrease in anxiety
Adhikari et al [Bibr R21][22]	Pre- post- intervention	NASC-CDM	N=19	23.4% decrease in anxiety 77.4 (SD 12.5 vs 59.3, SD 15.9; *P*<.001)
Cobbett et al [Bibr R22][23]	Intervention vs control	NASC-CDM^b^	N=56 Intervention=27	Intervention: anxiety (mean 73.26, SD 19.95, SE mean 3.84) Control: anxiety (mean 57.75, SD 15.25, SE mean 2.88)
Jung et al [24][Bibr R23]	Intervention versus control; Pre vs post test	STAI	n=60 Intervention =38	Intervention: pretest (mean 41.63, SD 9.30) and posttest (mean 39.52, SD 10.05) *P*=.29 Control: pretest (mean 46.97, SD 11.99) and posttest (mean 39.26, SD 10.90) *P*=.004

a STAI: State–Trait Anxiety Inventory

b NASC-CDM: Nursing Anxiety and Self-Confidence with Clinical Decision-Making Scale

In total, 4 studies reported decreased anxiety levels after the XR intervention. In [20], participant feedback supported that AR-CPR could be effective for cognitive offloading, reduction in performer anxiety, and increase in performer confidence in the care delivered. In the case of [21], 68% of nursing and 58% of medical interns reported that the extended reality intervention significantly decreased their anxiety about occupational needle injury prevention, and also in [22] the participants reported a 23.4% decrease in anxiety. In addition, in [19] at baseline, there were no significant differences between the intervention and regular groups. However, at follow-up, the intervention group showed significantly higher agreement with statements about increased familiarity (83% vs 76%, *P*=.04), enhanced confidence (92% vs 74%, *P*=.001), and reduced anxiety (93% vs 75%, *P*=.002). The VR intervention effectively improved familiarity, boosted confidence, and reduced anxiety in tracheostomy-related skills compared to the regular training.

One study [24] reported no statistical differences among the 3 groups. State anxiety and VAS for anxiety decreased in all groups after venipuncture.

A total of 2 studies reported higher anxiety in the intervention group. In [18] the anxiety score of the 3 groups decreased from pre-intervention to post-intervention (VR group: 59.14 (SD 9.62) to 56.72 (7.50); simulation group: 66.46 (8.60) to 57.65 (SD 10.35); and control group: 59.50 (8.35) to 57.65 (6.86). However, the VR group did not show significant improvement compared to the simulation and control groups, with the highest difference observed in the simulation one.

Also, anxiety scores were higher for students in the VR clinical simulation than for those in the face-to-face simulation [23]. However, the new technology rather than the VR simulation itself might have caused the increased anxiety. In addition, students were more comfortable with the mannequin, as they had previous exposure to this type of exercise.

#### Authors’ Opinions and Main Limitations of the Studies

The authors consider that the studies provided improvement in knowledge [18], demonstrated feasibility [19], improved educational quality [20], presented a promising pedagogical approach [22], and were educationally and cost-effective, as they allowed for repeated simulations [23]. This characteristic among others, contributes to the reduction of anxiety [24].

Nevertheless, even if the results were positive, all the studies included in this review performed a small sample, single-school or medical center, single-country sessions, which is a significant limitation as it is not very representative. The authors suggest using larger sample sizes with random selection to increase generalizability and conducting multicenter studies.

Furthermore, in 3 studies, the evaluation method was qualitative and self-reported, which might also lead to report bias [19-21]. In addition, the long-term effect was not evaluated in any of the articles and often the evaluation was only made at one-time point [22].

Several limitations were related to the use of new technologies, in the study by [23], students preferred face-to-face simulations, citing the similarities to practicing in a “real” situation and the immediate debriefing. Students who did not like the VR clinical simulation often cited technological issues, such as “online program was slow”, or “didn’t know where to find things. Similarly, in [24] they noted that the results might have been affected by the students’ IT expertise, while [18] pointed out that the unfamiliar VR environment could be a factor. In [22], they suggested that students more comfortable with technology might have been more willing to participate in the experiments.

An orientation activity built into the study design would be useful so that when the XR scenario is presented, students will not be focused on learning the software. Furthermore, the studies were often performed in a single session; multiple sessions would increase students’ familiarity with the tools. On the other hand [18], suggests that it would be better to establish an MR environment, using hand tracking and physically practicing the skills, rather than using the HMD controllers. This approach would also reduce the learner’s burden owing to unfamiliar environments, enabling experiences similar to reality.

Finally, none of the studies they engaged with the focus group (nurses and midwives) to identify and prioritize their real needs. A better approach would have been to perform a systematic conception method to translate their needs into tangible project objectives and features aligned with user expectations, rather than imposing the technological approach without working and thinking about the solution with them first.

## Discussion

### Limitations

The systematic review had several limitations. The research results could vary depending on the databases, languages, or types of articles included. Furthermore, the review focused on the use of extended reality for gesture simulation to reduce anxiety. Not many articles were found on this specific topic, highlighting a research gap. The results would differ if we included the use of these tools in other procedures, such as the management of medical situations or the treatment of anxiety with calming scenarios.

### Future Research Directions

This systematic review was conducted in preparation for a follow-up study on XR gesture simulation educational training for midwifery students’ anxiety management in the Midwifery Department at Grenoble Alpes University during the next academic year. The limited articles found in this review and their positive results highlight the research gap and underscore the importance of further exploring gesture simulation using XR tools to reduce student anxiety. Furthermore, the limitations found in the studies could be addressed by implementing a long-term follow-up with questionnaires, choosing a larger sample size, including diversity by testing in different centers, using MR, or engaging with the focus group.

The next intended study will focus on MR gesture simulation in an educational midwifery or nursing procedure. Collaboration with educators and users is essential to make an appropriate demonstrator, and the use a pre and post-test evaluation with diverse, evenly distributed participants from different centers and countries, to measure anxiety levels will be evaluated.

The first demonstrator prototype will be as follows:

1. Learning object: The learning object will be defined later on the development of the project, after discussing with the educators and students of the midwifery course.
2. Study design: RCT could be the primary methodology as it provides a more robust evidence by minimizing bias.
3. Participants: All of the 147 students at the Midwifery Department of the Grenoble Alpes University will be invited to participate in the study. The exclusion criteria will be incomplete training or questionnaires. Furthermore, depending on the learning object, only the promotion following the specific course will be included in the study, excluding the rest of the students. A posterior international demonstrator will be performed to validate the results obtained.
4. Device: To interact with the VR environment, Microsoft HoloLens 2 will be used, as the goal is to develop an MR tool. By seeing their hands, the students can physically practice the gestures to simulate and learn the procedure.
5. Anxiety Assessment Methods, Variables: Before and after the intervention, anxiety will be evaluated with the NASC-CDM [25,26*],* as it is specially designed for nursing students to measure stress and anxiety related to clinical procedures and academic responsibilities. n addition, slight modifications specific to our learning object will be added to the questionnaire.

### Conclusions

The results of the systematic review encourage the development of an extended reality gesture or procedure simulation system to evaluate and manage anxiety for nurses and midwives. Positive outcomes have been achieved as an improved learning experience, educational effectiveness, feasibility examples, and anxiety reduction. However, the search revealed limited research addressing this issue globally and a potential gap in the MR field, since it has not been used in any of the studies, and could meet the requirements and needs of midwifery and nursing students.

Furthermore, there were some limitations to the systematic review, including variability in results due to database selection and a narrow focus on gesture simulation. Expanding the scope to include other uses of XR, such as in medical procedures or calming scenarios could provide broader insights.

## Supplementary material

10.2196/68984 Checklist 1 PRISMA (Preferred Reporting Items for Systematic Reviews and Meta-Analyses) Checklist

## Abbreviations

ANESF: National French Association of Midwifery Students
AR: augmented reality
AR-CPR: augmented reality cardiopulmonary resuscitation
GAD-7: General Anxiety Disorder-7
MeSH: Medical Subject Heading
MR: mixed reality
NASC-CDM: Nursing Anxiety and Self-Confidence with Clinical Decision-Making Scale
PICO: population, intervention, control, and outcomes
PRISMA: Preferred Reporting Items for Systematic Reviews and Meta-Analyses
RTCs: Randomized Controlled Trials
STAI: State-Trait Anxiety Inventory
VR: virtual reality
XR: extended reality
